# Supplementary material for: Functional optimization of light-activatable Opto-GPCRs: Illuminating the importance of the proximal C-terminus in G-protein specificity
Source: Front Cell Dev Biol. 2023 Mar 1;11:1053022. doi: 10.3389/fcell.2023.1053022 (PMC10014536; doi:10.3389/fcell.2023.1053022)
Supplement: Supplementary file 2 [file DataSheet1.PDF]

## Sequences

**<u>Melanopsin-mGluR6</u>**

Mela(trunc)

MNPPSGPRVPPSPTQEPSCMATPAPPSWWDSSQSSISSLGRLPSISPTAPGTWAA
AWVPLPTVDVPDHAHYTLGTVILLVGLTGMLGNLTVIYTFCRSRSLRTPANMFIINLA
VSDFLMSFTQAPVFFTSSLYKQWLFGETGCEFYAFCGALFGISSMITLTAIALDRYLV
ITRPLATFGVASKRRAAFVLLGVWLYALAWSLPPFFGWSAYVPEGLLTSCSWDYMS
FTPAVRAYTMLLCCFVFFLPLLIIIYCYIFIFRAIRETGRALQTFGACKGNGESLWQRQ
RLQSECKMAKIMLLVILLFVLSWAPYSAVALVAFAGYAHVLTPYMSSVPAVIAKASAI
HNPIIYAITHPKYRVAIAQHLPCLGVLLGVSRRHSRPYPSYRSTHRSTLTSHTSNLHP
EQNVQKRKRSLKATSTVAAPPKGEDAEAHK

Mela(palm)

MNPPSGPRVPPSPTQEPSCMATPAPPSWWDSSQSSISSLGRLPSISPTAPGTWAA
AWVPLPTVDVPDHAHYTLGTVILLVGLTGMLGNLTVIYTFCRSRSLRTPANMFIINLA
VSDFLMSFTQAPVFFTSSLYKQWLFGETGCEFYAFCGALFGISSMITLTAIALDRYLV
ITRPLATFGVASKRRAAFVLLGVWLYALAWSLPPFFGWSAYVPEGLLTSCSWDYMS
FTPAVRAYTMLLCCFVFFLPLLIIIYCYIFIFRAIRETGRALQTFGACKGNGESLWQRQ
RLQSECKMAKIMLLVILLFVLSWAPYSAVALVAFAGYAHVLTPYMSSVPAVIAKASAI
HNPIIYAITHPKYRVAIAQHLPCLFHPEQNVQKRKRSLKATSTVAAPPKGEDAEAHK

Mela(trunc+IL3)

MNPPSGPRVPPSPTQEPSCMATPAPPSWWDSSQSSISSLGRLPSISPTAPGTWAA
AWVPLPTVDVPDHAHYTLGTVILLVGLTGMLGNLTVIYTFCRSRSLRTPANMFIINLA
VSDFLMSFTQAPVFFTSSLYKQWLFGETGCEFYAFCGALFGISSMITLTAIALDRYLV
ITRPLATFGVASKRRAAFVLLGVWLYALAWSLPPFFGWSAYVPEGLLTSCSWDYMS
FTPAVRAYTMLLCCFVFFLPLLIIIYCYIFIFRAIRETGRGVPETFNEAKGNGESLWQR
QRLQSECKMAKIMLLVILLFVLSWAPYSAVALVAFAGYAHVLTPYMSSVPAVIAKASA
IHNPIIYAITHPKYRVAIAQHLPCLGVLLGVSRRHSRPYPSYRSTHRSTLTSHTSNLHP
EQNVQKRKRSLKATSTVAAPPKGEDAEAHK

Mela(palm+IL3)

MNPPSGPRVPPSPTQEPSCMATPAPPSWWDSSQSSISSLGRLPSISPTAPGTWAA
AWVPLPTVDVPDHAHYTLGTVILLVGLTGMLGNLTVIYTFCRSRSLRTPANMFIINLA
VSDFLMSFTQAPVFFTSSLYKQWLFGETGCEFYAFCGALFGISSMITLTAIALDRYLV
ITRPLATFGVASKRRAAFVLLGVWLYALAWSLPPFFGWSAYVPEGLLTSCSWDYMS
FTPAVRAYTMLLCCFVFFLPLLIIIYCYIFIFRAIRETGRGVPETFNEAKGNGESLWQR
QRLQSECKMAKIMLLVILLFVLSWAPYSAVALVAFAGYAHVLTPYMSSVPAVIAKASA
IHNPIIYAITHPKYRVAIAQHLPCLFHPEQNVQKRKRSLKATSTVAAPPKGEDAEAHK

Mela(full CT+IL3)

MNPPSGPRVPPSPTQEPSCMATPAPPSWWDSSQSSISSLGRLPSISPTAPGTWAA
AWVPLPTVDVPDHAHYTLGTVILLVGLTGMLGNLTVIYTFCRSRSLRTPANMFIINLA
VSDFLMSFTQAPVFFTSSLYKQWLFGETGCEFYAFCGALFGISSMITLTAIALDRYLV
ITRPLATFGVASKRRAAFVLLGVWLYALAWSLPPFFGWSAYVPEGLLTSCSWDYMS
FTPAVRAYTMLLCCFVFFLPLLIIIYCYIFIFRAIRETGRGVPETFNEAKGNGESLWQR

QRLQSECKMAKIMLLVILLFVLSWAPYSAVALVAFAGYAHVLTPYMSSVPAVIAKASA
IHNPIIYALFHPEQNVQKRKRSLKATSTVAAPPKGEDAEAHK

Mela(palm+GRM6 short)

MNPPSGPRVPPSPTQEPSCMATPAPPSWWDSSQSSISSLGRLPSISPTAPGTWAA
AWVPLPTVDVPDHAHYTLGTVILLVGLTGMLGNLTVIYTFCRSRSLRTPANMFIINLA
VSDFLMSFTQAPVFFTSSLYKQWLFGETGCEFYAFCGALFGISSMITLTAIALDRYLV
ITRPLATFGVASKRRAAFVLLGVWLYALAWSLPPFFGWSAYVPEGLLTSCSWDYMS
FTPAVRAYTMLLCCFVFFLPLLIIIYCYIFIFRAIRETGRALQTFGACKGNGESLWQRQ
RLQSECKMAKIMLLVILLFVLSWAPYSAVALVAFAGYAHVLTPYMSSVPAVIAKASAI
HNPIIYAITHPKYRVAIAQHLPCAAPPKGEDAEAHK

**bRhod-b2AR**

bRhod-b2AR(trunc)

MNGTEGPNFYVPFSNKTGVVRSPFEAPQYYLAEPWQFSMLAAYMFLLIMLGFPINF
LTLYVTVQHKKLRTPLNYILLNLAVADLFMVFGGFTTTLYTSLHGYFVFGPTGCNLEG
FFATLGGEIALWSLVVLAIERYVVVCKPMSNFRFGENHAIMGVAFTWVMALACAAP
PLVGWSRYIPEGMQCSCGIDYYTPHEETNNESFVIYMFVVHFIIPLIVIFFCYGQLVFT
VKEAAAQQQESATTQKAEKEVTRMVIIMVIAFLICWLPYAGVAFYIFTHQGSDFGPIF
MTIPAFFAKTSAVYNPVIYIMMNKQFRNCMVTTLCCGKNPLGDDEASTYGNGYSSN
GNTGEQSGYHVEQEKENKLLCEDLPGTEDFVGHQGTVPSDNIDSQGRNCSTNDSL
L

bRhod-b2AR(palm)

MNGTEGPNFYVPFSNKTGVVRSPFEAPQYYLAEPWQFSMLAAYMFLLIMLGFPINF
LTLYVTVQHKKLRTPLNYILLNLAVADLFMVFGGFTTTLYTSLHGYFVFGPTGCNLEG
FFATLGGEIALWSLVVLAIERYVVVCKPMSNFRFGENHAIMGVAFTWVMALACAAP
PLVGWSRYIPEGMQCSCGIDYYTPHEETNNESFVIYMFVVHFIIPLIVIFFCYGQLVFT
VKEAAAQQQESATTQKAEKEVTRMVIIMVIAFLICWLPYAGVAFYIFTHQGSDFGPIF
MTIPAFFAKTSAVYNPVIYIMMNKQFRNCMVTTLCCLRRSSLKAYGNGYSSNGNTG
EQSGYHVEQEKENKLLCEDLPGTEDFVGHQGTVPSDNIDSQGRNCSTNDSLL

bRhod-b2AR(full CT)

MNGTEGPNFYVPFSNKTGVVRSPFEAPQYYLAEPWQFSMLAAYMFLLIMLGFPINF
LTLYVTVQHKKLRTPLNYILLNLAVADLFMVFGGFTTTLYTSLHGYFVFGPTGCNLEG
FFATLGGEIALWSLVVLAIERYVVVCKPMSNFRFGENHAIMGVAFTWVMALACAAP
PLVGWSRYIPEGMQCSCGIDYYTPHEETNNESFVIYMFVVHFIIPLIVIFFCYGQLVFT
VKEAAAQQQESATTQKAEKEVTRMVIIMVIAFLICWLPYAGVAFYIFTHQGSDFGPIF
MTIPAFFAKTSAVYNPVIYCRSPDFRIAFQELLCLRRSSLKAYGNGYSSNGNTGEQS
GYHVEQEKENKLLCEDLPGTEDFVGHQGTVPSDNIDSQGRNCSTNDSLL

bRhod-b2AR(IL3)

NGTEGPNFYVPFSNKTGVVRSPFEAPQYYLAEPWQFSMLAAYMFLLIMLGFPINFLT
LYVTVQHKKLRTPLNYILLNLAVADLFMVFGGFTTTLYTSLHGYFVFGPTGCNLEGF
FATLGGEIALWSLVVLAIERYVVVCKPMSNFRFGENHAIMGVAFTWVMALACAAPPL
VGWSRYIPEGMQCSCGIDYYTPHEETNNESFVIYMFVVHFIIPLIVIFFCYGRVFQEA
KRQLQKIDKSEGRFHVQNLSQVEQDGRTGHGLRRSSKFCLKEHKALMVIIMVIAFLI
CWLPYAGVAFYIFTHQGSDFGPIFMTIPAFFAKTSAVYNPVIYIMMNKQFRNCMVTT
LCCGKNPLGDDEASTTVSKTETSQVAPA

bRhod-b2AR(trunc+IL3)

MNGTEGPNFYVPFSNKTGVVRSPFEAPQYYLAEPWQFSMLAAYMFLLIMLGFPINF
LTLYVTVQHKKLRTPLNYILLNLAVADLFMVFGGFTTTLYTSLHGYFVFGPTGCNLEG
FFATLGGEIALWSLVVLAIERYVVVCKPMSNFRFGENHAIMGVAFTWVMALACAAP
PLVGWSRYIPEGMQCSCGIDYYTPHEETNNESFVIYMFVVHFIIPLIVIFFCYGRVFQ
EAKRQLQKIDKSEGRFHVQNLSQVEQDGRTGHGLRRSSKFCLKEHKALMVIIMVIAF
LICWLPYAGVAFYIFTHQGSDFGPIFMTIPAFFAKTSAVYNPVIYIMMNKQFRNCMVT
TLCCGKNPLGDDEASTYGNGYSSNGNTGEQSGYHVEQEKENKLLCEDLPGTEDFV
GHQGTVPSDNIDSQGRNCSTNDSLL

bRhod-b2AR(palm+IL3)

MNGTEGPNFYVPFSNKTGVVRSPFEAPQYYLAEPWQFSMLAAYMFLLIMLGFPINF
LTLYVTVQHKKLRTPLNYILLNLAVADLFMVFGGFTTTLYTSLHGYFVFGPTGCNLEG
FFATLGGEIALWSLVVLAIERYVVVCKPMSNFRFGENHAIMGVAFTWVMALACAAP
PLVGWSRYIPEGMQCSCGIDYYTPHEETNNESFVIYMFVVHFIIPLIVIFFCYGRVFQ
EAKRQLQKIDKSEGRFHVQNLSQVEQDGRTGHGLRRSSKFCLKEHKALMVIIMVIAF
LICWLPYAGVAFYIFTHQGSDFGPIFMTIPAFFAKTSAVYNPVIYIMMNKQFRNCMVT
TLCCLRRSSLKAYGNGYSSNGNTGEQSGYHVEQEKENKLLCEDLPGTEDFVGHQG
TVPSDNIDSQGRNCSTNDSLL

bRhod-b2AR(TM5+TM6)

MNGTEGPNFYVPFSNKTGVVRSPFEAPQYYLAEPWQFSMLAAYMFLLIMLGFPINF
LTLYVTVQHKKLRTPLNYILLNLAVADLFMVFGGFTTTLYTSLHGYFVFGPTGCNLEG
FFATLGGEIALWSLVVLAIERYVVVCKPMSNFRFGENHAIMGVAFTWVMALACAAP
PLVGWSRYIPEGMQCSCGIDYYTPHEETNNESFVIYMFVVHFIIPLIVIFFCYGQLVQ
EAKRQLQKIDKSEQESSKFCLKEKEVTRMVIIMVIAFLICWLPYAGVAFYIFTHQGSD
FGPIFMTIPAFFAKTSAVYNPVIYIMMNKQFRNCMVTTLCCGKNPLGDDEASTTVSK
TETSQVAPA

bRhod-b2AR(IL3 loop)

MNGTEGPNFYVPFSNKTGVVRSPFEAPQYYLAEPWQFSMLAAYMFLLIMLGFPINF
LTLYVTVQHKKLRTPLNYILLNLAVADLFMVFGGFTTTLYTSLHGYFVFGPTGCNLEG
FFATLGGEIALWSLVVLAIERYVVVCKPMSNFRFGENHAIMGVAFTWVMALACAAP
PLVGWSRYIPEGMQCSCGIDYYTPHEETNNESFVIYMFVVHFIIPLIVIFFCYGQLVFT
VKEAAAQQGRFHVQNLSQVEQDGRTGHGLRRSSATTQKAEKEVTRMVIIMVIAFLI
CWLPYAGVAFYIFTHQGSDFGPIFMTIPAFFAKTSAVYNPVIYIMMNKQFRNCMVTT
LCCGKNPLGDDEASTTVSKTETSQVAPA

bRhod-b2AR(IL2)

MNGTEGPNFYVPFSNKTGVVRSPFEAPQYYLAEPWQFSMLAAYMFLLIMLGFPINF
LTLYVTVQHKKLRTPLNYILLNLAVADLFMVFGGFTTTLYTSLHGYFVFGPTGCNLEG
FFATLGGEIALWSLVVLAIDRYFAITSPFKYQSLLTKNKAAIMGVAFTWVMALACAAP
PLVGWSRYIPEGMQCSCGIDYYTPHEETNNESFVIYMFVVHFIIPLIVIFFCYGQLVFT
VKEAAAQQQESATTQKAEKEVTRMVIIMVIAFLICWLPYAGVAFYIFTHQGSDFGPIF
MTIPAFFAKTSAVYNPVIYIMMNKQFRNCMVTTLCCGKNPLGDDEASTTVSKTETSQ
VAPA

bRhod-b2AR(IL2+IL3)

MNGTEGPNFYVPFSNKTGVVRSPFEAPQYYLAEPWQFSMLAAYMFLLIMLGFPINF
LTLYVTVQHKKLRTPLNYILLNLAVADLFMVFGGFTTTLYTSLHGYFVFGPTGCNLEG
FFATLGGEIALWSLVVLAIDRYFAITSPFKYQSLLTKNKAAIMGVAFTWVMALACAAP
PLVGWSRYIPEGMQCSCGIDYYTPHEETNNESFVIYMFVVHFIIPLIVIFFCYGRVFQ
EAKRQLQKIDKSEGRFHVQNLSQVEQDGRTGHGLRRSSKFCLKEHKALMVIIMVIAF
LICWLPYAGVAFYIFTHQGSDFGPIFMTIPAFFAKTSAVYNPVIYIMMNKQFRNCMVT
TLCCGKNPLGDDEASTTVSKTETSQVAPA

bRhod-b2AR(IL2+IL3+palm)

MNGTEGPNFYVPFSNKTGVVRSPFEAPQYYLAEPWQFSMLAAYMFLLIMLGFPINF
LTLYVTVQHKKLRTPLNYILLNLAVADLFMVFGGFTTTLYTSLHGYFVFGPTGCNLEG
FFATLGGEIALWSLVVLAIDRYFAITSPFKYQSLLTKNKAAIMGVAFTWVMALACAAP
PLVGWSRYIPEGMQCSCGIDYYTPHEETNNESFVIYMFVVHFIIPLIVIFFCYGRVFQ
EAKRQLQKIDKSEGRFHVQNLSQVEQDGRTGHGLRRSSKFCLKEHKALMVIIMVIAF
LICWLPYAGVAFYIFTHQGSDFGPIFMTIPAFFAKTSAVYNPVIYIMMNKQFRNCMVT
TLCLRRSSLKAYGNGYSSNGNTGEQSGYHVEQEKENKLLCEDLPGTEDFVGHQGT
VPSDNIDSQGRNCSTNDSLL
